# Prevalence and Radiographic Patterns of Third Molar Impaction and Agenesis in the Kazakh Population

**DOI:** 10.1016/j.identj.2025.100897

**Published:** 2025-07-30

**Authors:** Yasin Yasa, Kubeisin Altynbekov, Buse Serenay Kalafatoğlu, Shayakhmetova Meiramkul, Mohammad Alhusain, Chingis Altynbekov

**Affiliations:** a Department of Oral and Maxillofacial Radiology, Faculty of Dentistry, Ordu University, Ordu, Turkey; b Department of Orthopedic Dentistry, Faculty of Dentistry, Asfendiyarov Kazak National Medical University, Almaty, Kazakhstan; c Hollywood Smile Dental Clinic, Dubai

**Keywords:** Third molars, Impacted, Agenesis, Kazakhstan, Prevalence

## Abstract

**Background:**

Third molars are the most commonly impacted teeth and frequently exhibit congenital absence (agenesis). Their eruption may be affected by jaw location, tooth angulation, and various demographic and genetic factors. There are no data on third molar status in the Kazakh population.

**Purpose:**

This study aimed to determine the prevalence of third molar impaction and agenesis in the Kazakh population, investigate radiographic patterns (eg, angulation, location, root morphology), and assess associated pathologies such as alveolar bone loss and caries.

**Study Design, Setting, Sample:**

This retrospective study evaluated 2306 panoramic radiographs from a private clinic in Almaty, Kazakhstan, collected between December 2020 and July 2024. Participants were 15 to 30 years of age, with no history of third molar extractions or poor-quality radiographs. A total of 9224 third molar sites were evaluated.

**Independent Variable:**

Age, sex and jaw location (maxilla vs mandible) were considered primary factors potentially influencing third molar impaction and agenesis.

**Main Outcome Variables:**

Key outcomes included the presence or absence of impacted and/or congenitally missing (agenesis) third molars, as well as radiographic characteristics (Winter’s classification, Pell–Gregory levels, root morphology).

**Covariates:**

Covariates encompassed eruption status (fully impacted, partially impacted, or erupted), tooth-specific location and associated pathologies.

**Analyses:**

Chi-square tests were used to compare prevalence rates and distribution patterns by sex, age group and tooth location. Significance was set at *P* < .05.

**Results:**

Impaction was detected in 68.0% of individuals aged 15 to 20. Agenesis occurred in 37.28%. Females had slightly higher rates of multiple impactions. Maxillary molars were predominantly completely impacted, whereas mandibular molars were mostly partially impacted. Alveolar bone loss (52.08%) and caries (36.9%) were the most frequent pathologies. Mesioangular impaction dominated in the mandible; distoangular positions prevailed in the maxilla.

**Conclusions:**

The high prevalence of third molar impaction and agenesis underscores the importance of region-specific clinical guidelines. Early radiographic evaluation may aid in preventing complications and improving outcomes in this population.

## Introduction

In the International Classification of Diseases by the World Health Organization, both impacted teeth and tooth agenesis are classified under developmental dental disorders. Impacted teeth are defined as those that remain partially or completely embedded within the jawbone and fail to erupt normally, often due to insufficient jaw size, abnormal tooth positioning, or anatomical/pathological obstructions. Clinically, impacted teeth can lead to complications such as pericoronitis, cyst formation and pressure on adjacent teeth. By contrast, tooth agenesis refers to the congenital absence of 1 or more teeth due to genetic mutations, environmental influences, or syndromic conditions such as ectodermal dysplasia. This condition can contribute to orthodontic malocclusions and impair chewing and speech functions.^1^

Third molars, the last teeth to erupt in the dental arch, exhibit significant developmental variability and are the most commonly impacted teeth, with impaction frequencies ranging from 3.8% to 70% across various populations.^2^, ^3^, ^4^, ^5^, ^6^, ^7^, ^8^, ^9^ Their chronological development typically begins between ages 7 and 9, with enamel formation completing between 12 and 16 years, and root development concluding between 18 and 25 years.^10^, ^11^, ^12^ Although the eruption timing varies across populations, third molars generally erupt between the ages of 17 and 21.^13^ While in some individuals all third molars develop and erupt normally, in others these teeth may never form or may remain impacted. This variability stems from the sensitivity of odontogenesis to genetic and environmental factors.

In modern dentistry, impacted third molars are frequently encountered clinical cases, arising when a tooth’s eruption is hindered or its path is altered – often due to insufficient jaw space, irregular dental alignment, or pathological conditions during development.^14^^,^^15^ However, third molars may not only remain impacted but also fail to develop altogether in some individuals. Third molar agenesis is a common dental developmental anomaly resulting from the interaction of genetic and environmental factors. Both impacted third molars, and third molar agenesis are important considerations for dentists, as they can significantly affect oral health, chewing function and orthodontic balance.^16^

Although numerous studies and approaches address the pathologies and treatments of third molars, examining demographic and regional factors is crucial to understanding the causes of impaction and its relationship with other dental anomalies. Hence, elucidating how impaction prevalence varies by geography and demographic characteristics is essential for identifying underlying aetiologies. Moreover, insights gained from such investigations significantly contribute to improving clinical decision-making and treatment planning. A comprehensive understanding of regional and population-based variations will ultimately enhance clinicians’ ability to provide more effective and personalised care in the management of third molars.

The high prevalence of impacted third molars, particularly in Asia and the Middle East, underscores the need for region-specific guidelines that consider unique demographic and anatomical factors influencing third molar development.^13^ However, no data are currently available on third molars in the Kazakh population. Therefore, the objective of this study was to determine the prevalence of impacted third molars and third molar agenesis in the Kazakh population, to analyse their radiographic patterns, including angulation, location, and associated pathologies, and to explore any differences by gender, as well as potential correlations between impaction and agenesis.

## Material and methods

This retrospective study was conducted by reviewing panoramic radiographs of patients who presented to a private clinic in Almaty, Kazakhstan, between December 2020 and July 2024 for various dental reasons. The study adhered to the principles outlined in the Declaration of Helsinki. Ethical approval was obtained from the Asfendiyarov Kazak National Medical University Clinical Research Ethics Committee (26 December 2024; approval number 30-251224). A total of 2306 panoramic radiographs were examined.

The inclusion criteria for this study encompassed patients aged between 15 and 30 years who had available panoramic radiographs and were healthy individuals with no previous surgical procedures involving the posterior quadrants of either jaw. Conversely, radiographs were excluded if they showed artefacts, contained low-quality images, belonged to patients with signs of orthodontic treatment, or were from patients who had undergone third molar extractions.

All panoramic radiographs were examined by an experienced oral and maxillofacial radiologist under standardised lighting conditions, using RadiAnt DICOM Viewer software. The following parameters were evaluated on each panoramic radiograph:

• Eruption status of the third molars.
• Pathological conditions associated with third molars.
• Congenital absence (agenesis) and impaction of third molars.
• Angular position of third molars based on Winter’s classification.
• Root morphology of the third molars.
• Relationship between mandibular third molars and the inferior alveolar canal (IAC).
• The eruption status of third molars was assessed using the Pell–Gregory classification.^17^
• Level A: The highest point of the third molar is at or above the occlusal plane of the adjacent second molar (erupted).
• Level B: The highest point of the third molar is below the occlusal plane but above the cervical line of the second molar (partially impacted).
• Level C: The highest point of the third molar is below the cervical line of the second molar (impacted).

Third molars were excluded from analysis if the adjacent second molar was absent or exhibited extensive crown resorption that prevented accurate assessment.

Pathologies associated with third molars and their radiographic diagnoses were defined as follows:

• Alveolar bone loss: The alveolar crest around the third molar is located 2 mm or more apically from the cementoenamel junction.
• Periapical radiolucency: A widened periodontal ligament space in the apical third of the root, potentially accompanied by abscess-cyst formation.
• Root resorption of the adjacent tooth: Inability to clearly trace the root boundaries of the tooth adjacent to the third molar.
• Caries on the adjacent tooth: A radiolucent area on the crown of the tooth adjacent to the third molar.
• Caries (third molar): Radiolucency observed on the crown or root of the third molar.

Dental agenesis was defined as the congenital absence of 1 or more primary or permanent teeth (excluding extracted teeth). The absence of third molar germs in panoramic radiographs of patients aged 15-20 years with no history of surgical removal was classified as third molar agenesis.

The angular positions of third molars were evaluated according to Winter’s classification,^18^ referring to the angle formed between the longitudinal axes of the second and third molars: Mesioangular, Distoangular, Vertical, Horizontal, Buccolingual, Inverted.

Third molars were excluded from angular analysis if the adjacent second molar was missing, or if the second molar’s longitudinal axis was not identifiable due to extensive crown or root resorption.

Root morphology of the third molars was categorised as curved, dilacerated and straight. Root dilaceration was identified when the angle between the long axis of the root and the dilacerated portion exceeded 90° in the mesiodistal plane. Roots with angles between 0° and 90° were considered curved, and those at 0° were classified as straight. Roots that were incompletely formed or severely resorbed were excluded from the root morphology evaluation.

For the dedicated assessment of the relationship between mandibular third molars and IAC, only teeth satisfying strict proximity criteria were retained. Mandibular third molars were included only when at least 1 of the 7 radiographic signs of close IAC proximity, originally proposed by Rood and Shehab,^19^ was observed: darkening of the root, deflected root, interruption of the white line, narrowing of the IAC, diversion of the IAC, dark and bifid root, narrowing of the root**.**

All statistical procedures were performed with IBM SPSS Statistics v26 (IBM Corp.). Categorical data are presented as frequencies and percentages. Associations between variables such as sex, age group, jaw location, eruption status, impaction type, root morphology and related pathologies were examined with Pearson’s χ^2^ test; Fisher’s exact test was applied. Statistical significance was set at *P* < .05.

To verify the consistency of the radiographic classifications, 15% of the images were re-evaluated by the same examiner after a 3-month interval. Agreement between the initial and repeat assessments was measured with Cohen’s κ, which exceeded 0.92 for all repeated measures, indicating excellent intra-observer reliability.

## Results

Demographic characteristics of the study participants are summarised in Table 1. The mean age and age group distributions were comparable between male and female participants. A total of 9224 third molar sites were evaluated from 2306 individuals.

**Table 1:** Demographic characteristics of participants.

	Age group 15-20	Age group 21-25	Age group 26-30	Total	Age
	*n* (%)	*n* (%)	*n* (%)	*n* (%)	Mean (SD)
Male	343 (41.78)	248 (30.21)	230 (28.01)	821 (100.0)	21.84 (4.57)
Female	604 (40.67)	480 (32.32)	401 (27.00)	1485 (100.0)	21.91 (4.46)
Total	947 (41.07)	728 (31.57)	631 (27.36)	2306 (100.0)	21.89 (4.49)

*n*, amount of individuals.

Table 2 presents the distribution of impacted teeth and agenesis by gender among 947 individuals aged 15 to 20 years. Impacted teeth were present in 68.0% of all subjects, with a slightly higher prevalence in females (69.7%) compared to males (65.0%), though this difference was not statistically significant (*P* = .16). Regarding agenesis, 37.28% of all subjects exhibited this condition, with similar rates between males (38.2%) and females (36.8%), showing no significant gender-based difference (*P* = .71). The distribution of the number of impacted teeth revealed a statistically significant difference between genders (*P* = .0013), with females showing higher prevalence of multiple impactions – notably, 7.8% of females had 3 impacted teeth (compared to 2.9% of males) and 6.1% had 4 impacted teeth (vs 2.3% of males). In contrast, the distribution pattern for the number of third molar agenesis sites showed no significant gender differences (*P* = .91), with comparable patterns observed across both genders for single and multiple tooth agenesis.

**Table 2:** Distribution of impacted third molars and third molar agenesis sites by gender in individuals aged 15-20 years.

	Male	Female	Total	*P* value
	*n* (%)	*n* (%)	*n* (%)	
**Impacted teeth**				
Present	223 (65.0)	421 (69.7)	644 (68.0)	.16
Absent	120 (35.0)	183 (30.3)	303 (32.0)	
**Agenesis**				
Present	131 (38.2)	222 (36.8)	353 (37.28)	.71
Absent	212 (61.8)	382 (63.2)	594 (62.72)	
**Number of impacted teeth**				
0 teeth	120 (35.0)	183 (30.3)	303 (32.00)	.0013
1 tooth	71 (20.7)	106 (17.6)	177 (18.69)	
2 teeth	134 (39.1)	231 (38.3)	365 (38.54)	
3 teeth	10 (2.9)	47 (7.8)	57 (6.02)	
4 teeth	8 (2.3)	37 (6.1)	45 (4.75)	
**Number of third molar agenesis sites**				
0 teeth	212 (61.8)	382 (63.3)	594 (62.72)	.91
1 tooth	46 (13.4)	80 (13.3)	126 (13.31)	
2 teeth	37 (10.8)	70 (11.6)	107 (11.30)	
3 teeth	21 (6.1)	30 (5.0)	51 (5.39)	
4 teeth	27 (7.9)	42 (7.0)	69 (7.29)	
Total	343 (36.2)	604 (63.8)	947 (100)	

*n*, amount of individuals; *P* values derived from χ^2^ tests.

The analysis of third molar eruption status revealed significant variations by location, gender and age group (Table 3). Maxillary teeth exhibited higher rates of impaction (42.3% and 41.2%, respectively) compared to the mandible. Conversely, partially impacted molars were more prevalent in the mandible (80%) than the maxilla (20%). Overall, 21.5% of third molars were impacted, 22.11% partially impacted, 27.35% erupted and 29.04% were missing or agenesis. Gender disparities were notable, with females showing higher rates of missing/agenesis third molars (30.7% vs 26.1% in males, *P* < .001) and impacted molars (22.7% vs 19.4%, *P* < .001). Age stratification demonstrated a decline in impaction rates with increasing age: 63.4% in the 15 to 20 years group, 22.1% in the 21-25 years group and 14.4% in the 26 to 30 years group (*P* < .001). These findings underscore the influence of anatomical location, gender and age on third molar eruption patterns, with statistically significant differences across all categories (*P* < .001).

**Table 3:** Distribution of eruption status of teeth by gender, age group, location and teeth.

	Impacted	Partially impacted	Erupted	Missing/Agenesis	Total	*P* value
	*n* (%)	*n* (%)	*n* (%)	*n* (%)	*n* (%)	
**Gender**						
Female	1346 (22.7)	1301 (21.9)	1471 (24.8)	1822 (30.7)	5940 (100)	<.001
Male	637 (19.4)	738 (22.5)	1052 (32.0)	857 (26.1)	3284 (100)	
**Age group**						
15-20 years	1258 (63.4)	1146 (56.2)	615 (24.4)	769 (28.7)	3788 (41.07)	<.001
21-25 years	439 (22.1)	579 (28.4)	1012 (40.1)	882 (32.9)	2912 (31.57)	
26-30 years	286 (14.4)	314 (15.4)	896 (35.5)	1028 (38.4)	2524 (27.36)	
**Location**						
18	839 (42.3)	165 (8.1)	636 (25.2)	666 (24.9)	2306 (25.0)	
28	818 (41.2)	242 (11.9)	599 (23.7)	647 (24.1)	2306 (25.0)	
Total (maxilla)	1657 (83.6)	407 (20.0)	1235 (49.0)	1313 (49.0)	4612 (50.0)	
38	173 (8.7)	806 (39.5)	635 (25.2)	692 (25.8)	2306 (25.0)	<.001
48	153 (7.7)	826 (40.5)	653 (25.9)	674 (25.2)	2306 (25.0)	
Total (mandible)	326 (16.4)	1632 (80.0)	1288 (51.0)	1366 (51.0)	4612 (50.0)	-
Total	1983 (21.50)	2039 (22.11)	2523 (27.35)	2679 (29.04)	9224 (100)	-

*n*, amount of individuals; *P* values derived from χ^2^ tests for categorical variables.

Analysis of tooth eruption status revealed significant differences across gender, age group and location (*P* < .001). Of the 9224 third molar sites examined, 29.04% were missing or presented with agenesis, 27.35% were fully erupted, 22.11% were partially impacted, and 21.50% were completely impacted. Gender distribution showed that females represented a higher proportion of cases in all eruption categories except for fully erupted teeth, with particularly pronounced differences in the completely impacted (67.9% female vs 32.1% male) and missing/agenesis (68.0% female vs 32.0% male) categories. Age-related patterns demonstrated that the youngest age group (15-20 years) had the highest prevalence of completely impacted teeth (63.4%), while the oldest age group (26-30 years) showed the highest proportion of missing teeth (38.4%). Regarding location, maxillary teeth accounted for the vast majority of completely impacted teeth (83.6%), with teeth 18 and 28 being the most frequently affected (42.3% and 41.2%, respectively). Conversely, mandibular teeth represented 80.0% of all partially impacted teeth, predominantly affecting teeth 38 and 48 (39.5% and 40.5%, respectively).

Among the 1252 pathological findings recorded (Table 4), alveolar bone loss was by far the most frequent lesion (52.08%), followed by caries of the third molar (36.90%), while apical radiolucencies, root resorption in the adjacent tooth, and distal caries in the second molar were relatively uncommon. The distribution of lesions did not differ significantly between females and males (*P* = .62); both sexes displayed a similar pattern of pathologies, with alveolar bone loss accounting for roughly half of all findings. Pathology frequency varied markedly by eruption category (*P* < .001). Almost all lesions (85.9%) occurred around fully erupted third molars, whereas impacted teeth – although representing only 2.2% of the sample – harboured a disproportionate share of apical radiolucencies (10.3% of all such lesions) and root resorption of the adjacent tooth (42.9%). Partially erupted teeth contributed the highest proportion of distal caries in the second molar (58.2% within the partially erupted category). Maxillary third molars accounted for most alveolar bone-loss cases, whereas mandibular molars were responsible for the majority of caries and apical radiolucencies; these location-based differences were highly significant (*P* < .001).

**Table 4:** Distribution of pathologies associated with third molars by gender, eruption status and location.

	Alveolar bone loss	Apical radiolucencies	Root resorption in adjacent tooth	Caries in adjacent tooth	Caries	Total	*P* value
**Gender**	*n* (%)	*n* (%)	*n* (%)	*n* (%)	*n* (%)	*n* (%)	
Female	342 (50.15%)	17 (2.49%)	22 (3.23%)	39 (5.72%)	262 (38.42%)	682 (100%)	.62
Male	310 (54.39%)	12 (2.11%)	20 (3.51%)	28 (4.91%)	200 (35.09%)	570 (100%)	
**Eruption status**							
Impacted	0 (0.00)	3 (10.34)	18 (42.86)	7 (10.45)	0 (0.00)	28 (2.24)	
Partially impacted	30 (4.6)	12 (41.38)	17 (40.48)	39 (58.21)	50 (10.82)	148 (11.83)	<.001
Erupted	622 (95.4)	14 (48.28)	7 (16.67)	21 (31.34)	412 (89.18)	1076 (85.93)	
**Location**							
18	202 (30.98)	4 (13.79)	13 (30.95)	9 (13.43)	112 (24.24)	340 (27.16)	
28	194 (29.75)	3 (10.34)	4 (9.52)	3 (4.48)	92 (19.91)	296 (23.64)	<.001
Maxilla (total)	396 (31.63%)	7 (0.56%)	17 (1.36%)	12 (0.96%)	204 (16.29%)	636 (50.80%)	
38	118 (18.10)	9 (31.03)	13 (30.95)	23 (34.33)	140 (30.30)	303 (24.20)	
48	138 (21.17)	13 (44.83)	12 (28.57)	32 (47.76)	118 (25.54)	313 (25.00)	
Mandible (total)	256 (20.45%)	22 (1.76%)	25 (2.00%)	55 (4.39%)	258 (20.61%)	616 (49.20%)	
Total	652 (52.08%)	29 (2.32%)	42 (3.35%)	67 (5.35%)	462 (36.90%)	1252 (100%)	

*n*, amount of individuals; *P* values derived from χ^2^ tests for categorical variables.

The angular positions of third molar teeth were found to be statistically significant in relation to eruption status and tooth position (*P* < .001) (Table 5). Of the 6545 teeth examined, the most common angulation was mesioangular (45.23%), followed by distoangular (37.07%) and vertical (12.54%). Gender analysis revealed that females had a higher prevalence of mesioangular (46.4% vs 43.2% in males) and distoangular (37.9% vs 35.7% in males) angulations, while males showed a higher frequency of vertical angulation (14.9% vs 11.1% in females). Regarding eruption status, partially erupted teeth were predominantly associated with mesioangular (46.59%) and horizontal (61.26%) angulations, while impacted teeth showed the highest prevalence of distoangular angulation (46.75%). Analysis by location demonstrated marked differences between maxillary and mandibular teeth, with maxillary teeth predominantly exhibiting distoangular angulation (98.10% of all distoangular teeth), while mandibular teeth showed a higher prevalence of mesioangular (91.08%), horizontal (94.47%) and buccolingual (84.42%) angulations.

**Table 5:** Distribution of third molar angulation patterns by gender, eruption status and location.

	Buccolingual	Distoangular	Mesioangular	Horizontal	Inversion	Vertical	Total	*P* value
	*n* (%)	*n* (%)	*n* (%)	*n* (%)	*n* (%)	*n* (%)	*n* (%)	
**Gender**								
Male	23 (0.95)	867 (35.7)	1049 (43.2)	125 (5.1)	1 (0.04)	362 (14.9)	2427 (37.08)	
Female	54 (1.3)	1559 (37.9)	1911 (46.4)	128 (3.1)	7 (0.17)	459 (11.1)	4118 (62.92)	<.001
**Eruption status**								
Impacted	36 (46.75)	1066 (43.94)	463 (15.64)	50 (19.76)	8 (100.0)	360 (43.85)	1983 (30.3)	
Partially erupted	40 (51.95)	360 (14.84)	1379 (46.59)	155 (61.26)	0 (0.0)	105 (12.79)	2039 (31.15)	<.001
Fully erupted	1 (1.30)	1000 (41.23)	1118 (37.77)	48 (18.97)	0 (0.0)	356 (43.36)	2523 (38.55)	
**Location**								
18	5 (6.49)	1165 (48.02)	133 (4.49)	9 (3.56)	5 (62.5)	323 (39.34)	1640 (25.06)	
28	7 (9.09)	1215 (50.08)	131 (4.43)	5 (1.98)	2 (25.0)	299 (36.42)	1659 (25.35)	
Maxilla (total)	12 (15.58)	2380 (98.10)	264 (8.92)	14 (5.53)	7 (87.5)	622 (75.76)	3299 (50.40)	
38	40 (51.95)	24 (0.99)	1337 (45.17)	106 (41.90)	1 (12.5)	106 (12.91)	1614 (24.66)	<.001
48	25 (32.47)	22 (0.91)	1359 (45.91)	133 (52.57)	0 (0.0)	93 (11.33)	1632 (24.94)	
Mandible (total)	65 (84.42)	46 (1.90)	2696 (91.08)	239 (94.47)	1 (12.5)	199 (24.24)	3246 (49.60)	
Total	77 (1.18)	2426 (37.07)	2960 (45.23)	253 (3.87)	8 (0.12)	821 (12.54)	6545 (100)	

*n*, number of third molar sites; *P* values derived from χ^2^ tests for categorical variables.

Analysis of root morphology patterns revealed significant differences across eruption status and location (*P* < .001), while no significant gender differences were observed (*P* = .870) (Table 6). Among the 4665 teeth examined, straight roots were the most prevalent morphology (66.26%), followed by curved roots (24.31%) and dilaceration (9.43%). Gender distribution showed nearly identical patterns, with males and females exhibiting similar proportions of dilaceration (9.36% vs 9.47%), straight roots (66.70% vs 65.98%) and curved roots (23.93% vs 24.55%). Regarding eruption status, fully erupted teeth had the highest prevalence of dilaceration (63.41%), while partially impacted teeth showed the highest frequency of curved roots (34.22%). Anatomical location analysis demonstrated marked differences between maxillary and mandibular teeth, with maxillary teeth predominantly exhibiting straight root morphology (62.44% of all straight roots), while mandibular teeth showed significantly higher prevalence of dilaceration (70.45%) and curved roots (80.69%). Specifically, teeth 38 exhibited the highest frequency of dilaceration (36.14%) and curved roots (41.45%), while teeth 18 had the highest prevalence of straight roots (31.61%).

**Table 6:** Distribution of third molar root morphology patterns by gender, eruption status and location.

	Dilaceration	Straight	Curved	Total	*P* value
	*n* (%)	*n* (%)	*n* (%)	*n* (%)	
**Gender**					
Male	171 (9.36)	1218 (66.70)	437 (23.93)	1826 (39.14)	.870
Female	269 (9.47)	1873 (65.98)	697 (24.55)	2839 (60.86)	
**Eruption status**					
Impacted	25 (5.68)	671 (21.71)	73 (6.44)	769 (16.48)	
Partially impacted	136 (30.91)	890 (28.79)	388 (34.22)	1414 (30.31)	<.001
Erupted	279 (63.41)	1530 (49.5)	673 (59.35)	2482 (53.20)	
**Location**					
18	67 (15.23)	977 (31.61)	91 (8.02)	1135 (24.33)	
28	63 (14.32)	953 (30.83)	128 (11.29)	1144 (24.52)	<.001
Maxilla (total)	130 (29.55)	1930 (62.44)	219 (19.31)	2279 (48.85)	
38	159 (36.14)	558 (18.05)	470 (41.45)	1187 (25.45)	
48	151 (34.32)	603 (19.51)	445 (39.24)	1199 (25.70)	
Mandible (total)	310 (70.45)	1161 (37.56)	915 (80.69)	2386 (51.15)	
Total	440 (9.43)	3091 (66.26)	1134 (24.31)	4665 (100)	

*n*, number of third molar sites; *P* values derived from χ^2^ tests for categorical variables.

Out of the 2847 mandibular third molar teeth deemed suitable for evaluation, 886 (31.12%) were found to be in close radiographic proximity to the mandibular canal. Among these 886 teeth, the distribution of radiographic findings indicative of nerve proximity was as follows: darkening of the root in 309 teeth (34.87%), narrowing of the root in 60 teeth (6.77%), dark and bifid apex in 94 teeth (10.61 %), interruption of the white line of the canal in 61 teeth (6.89%), narrowing of the canal in 215 teeth (24.27%), deviation of the root in 77 teeth (7.90%) and diversion of the canal in 70 teeth (2.46%).

## Discussion

In this study evaluating the prevalence of third molar impaction and agenesis in the Kazakh population, we address an ongoing debate in the literature regarding variations in the development of third molars related to gender, jaw location and various patterns of third molars. The relative contributions of genetic and environmental factors to M3 agenesis remain unclear. While some authors argue that impaction and agenesis may share similar underlying etiological mechanisms, including genetic and environmental influences, others suggest these are fundamentally distinct phenomena.^20^ Given the scarcity of existing epidemiological data from Kazakhstan, our findings provide valuable insights into regional patterns of third molar development.

Various classification systems for impacted mandibular third molars have been developed in the literature. Initially, Winter^18^ classified third molars based on the angle between their longitudinal axis and the occlusal plane. This fundamental classification was later expanded to include buccoangular, linguoangular, inverted and unusual positions.^21^ The classification proposed by Pell and Gregory,^17^ which is widely used, is based on 2 different criteria: the relationship of the mandibular ramus to the second molar (classes I, II and III) and the depth of the tooth relative to the occlusal plane (positions A, B and C). This classification is still preferred in many contemporary studies.^5^^,^^22^, ^23^, ^24^ Peterson modified this system by incorporating the angle of the tooth.^25^ In addition, in clinical practice and for insurance purposes, an alternative classification based on the nature of the overlying tissue is used, categorising impactions as soft tissue impaction, partial bony impaction and complete bony impaction.^26^ However, while these traditional classifications aim to determine tooth position and extraction difficulty, they have certain limitations, such as using the occlusal plane as a reference point, which does not adequately assess relationships with the mandibular canal and lingual nerve. In this context, a new classification proposed by Juodzbalys and Daugela^27^ presents an approach based on anatomical and radiological findings. This system evaluates impacted third molars’ extraction difficulty by scoring their mesiodistal, apicocoronal, buccolingual and spatial positions. By utilising advanced imaging methods such as Cone‑Beam Computed Tomography, this classification aims to better predict the risk of inferior alveolar and lingual nerve injury, making it a more comprehensive and reliable guide for surgical planning than traditional classifications. Subsequently, new classifications have also been introduced. The classification proposed by Santosh Patil combines clinical and radiological findings, offering a system based on pathological changes that range from asymptomatic teeth to cases associated with malignant tumours. This system not only assesses the position of the tooth but also evaluates its pathological relationship with surrounding tissues.^28^ For comparing regional studies on impacted teeth, it is important to establish and follow a standardised approach. As our study is the first to be conducted in Kazakhstan, we chose to use Winter’s expanded classification and Pell and Gregory’s position classification, which has been the most widely preferred method for many years, to facilitate meaningful comparisons with studies from other regions.

In the literature, there is no standardised age range for assessing the prevalence of impacted teeth and third molar eruption. Some studies suggest that third molar eruption is generally completed between the ages of 17 and 20.^13^ In addition, the National Institute for Clinical Excellence^29^ (NICE) guidelines report that third molars typically erupt between 18 and 24 years of age, although eruption may occur much later in some cases. After eruption is completed, root growth continues for some time to achieve apical (root tip) closure. Complete closure of third molar root tips generally occurs around the age of 21 to 22 years.^30^^,^^31^ In our study, we conducted our evaluations of agenesis and of impacted third molars within the 15 to 20 age group, considering the likelihood of extraction after the age of 20. A review of the existing literature reveals significant differences in the age ranges used to evaluate the prevalence of impacted teeth.^13^ While some studies include broader age groups, it is observed that the prevalence of impacted teeth decreases, particularly after the 20s. This decrease is attributed to the natural completion of eruption of some third molars with advancing age, as well as to extractions performed due to pathological reasons or prophylactic interventions. According to current consensus, the highest prevalence of impacted teeth is observed in the 17 to 25 age range.^32^^,^^33^ This emphasises the importance of age-stratified analyses, as extractions or losses in older age groups may lead to an underestimation of the true prevalence of impacted teeth.

A systematic review conducted in 2024, based on data from 183,828 patients, examined the distribution of impacted third molar prevalence across geographical regions. This study reported that Asian populations had the highest prevalence of impacted teeth (43.1%), followed by Middle Eastern populations (36.5%). The prevalence of impacted teeth was found to be lowest (24.5%) in European populations**.** African (33.5%) and American (33%) populations showed intermediate levels of prevalence.^13^ In our study, the prevalence of impacted teeth in individuals aged 15 to 20 years was determined to be 68.0%. While this rate is consistent with the high prevalence reported in Asian populations, it is generally higher overall. When evaluating the influence of gender on the prevalence of impacted third molars, some studies suggest a slight female predisposition to third molar impaction.^34^^,^^35^ For instance, the study from Oman reported that 60% of females, compared to 40% of males, exhibited impaction, and other studies have similarly noted higher impaction rates in females.^32^ In our study, a statistically significant difference between genders was observed, with a slightly higher number of females presenting with 1 or more impacted third molars compared to males. Proposed explanations for this disparity include a smaller jaw size and an earlier cessation of skeletal growth in females, which may reduce the available space for erupting third molars. However, several studies, including 1 from Kerala, India,^34^ and studies by Alamri et al^5^ and Pedro et al^7^ have found no significant gender differences. These findings suggest that the gender disparity in third molar impaction might be population-specific or only modest in magnitude.

Carter and Worthington,^36^ in a systematic review and meta-analysis examining the distribution of agenesis across geographical regions, reported that Asian populations had the highest rate of agenesis (29.71%), while African populations showed the lowest rate (5.74%). The study indicated that third molar agenesis in Africa was significantly lower than all other geographical regions, although it was emphasised that this continent was inadequately sampled. In addition, European (21.60%), South American (18.19%) and North American (17.88%) populations were found to have significantly lower frequencies of third molar agenesis compared to Asian populations. In our study, the prevalence of dental agenesis in individuals aged 15-20 years was determined to be 37.28%. This rate is higher than the average agenesis rate (29.71%) reported in studies conducted in the Asian population.^36^ This difference can be explained by our study sample including a different age group (15-20 years) and the influence of regional/genetic factors. This aligns with the concept of an evolutionary reduction in the number of teeth; third molars are the most likely teeth to be absent from birth.^37^ The same meta-analysis reported that women had a 14.02% higher (5.38%-23.35%, *P* = .0014) rate of agenesis compared to men. Furthermore, our study found no significant difference between genders in terms of dental agenesis.

Findings in this study indicated that alveolar bone loss was the most common finding for the third molars (52.08), and evidence of alveolar bone loss on the distal aspect of the second molar was observed mostly in cases where the third molar was fully erupted (95%). In the study conducted in Poland,^37^ this rate was found to be 21.1%. Both studies demonstrated that alveolar bone loss was observed more frequently in males.

In our study, periapical radiolucencies were observed at the lowest frequency among the pathologies detected around impacted third molars (2.32%). Linden et al similarly reported an incidence of 2.2% in their study.^38^ However, this low incidence does not rule out the potential risks posed by these lesions. The literature suggests that even when these lesions occur infrequently, they may progress into large cysts or, albeit rarely, odontogenic tumours. Consequently, despite their asymptomatic nature, these radiolucent findings require careful evaluation and periodic monitoring to enable timely intervention should any malignant or expansive changes be suspected.

Our findings regarding root resorption of the adjacent tooth caused by third molars are similar to those reported by some studies in the literature (approximately 1%-3%).^24^^,^^37^^,^^39^ Although this phenomenon was also rarely observed, it can ultimately lead to the loss of the second molar if not detected early. In addition, in our study, there was no significant difference between partially or fully impacted teeth in terms of root resorption on the adjacent tooth. However, a study by Poszytek et al^37^ reported that particularly partially impacted, horizontally or mesially inclined third molars carry a higher risk for root resorption. Some studies have noted improvement in periodontal parameters on the second molar after third molar extraction.^40^

The presence of a third molar is a factor that increases caries formation on the distal surface of the second molar.^41^ In our study, the rate of second molar caries was found to be 5.35% for all pathologies. In the literature, a very wide range of results (3%-42%^3^^,^^24^^,^^33^^,^^37^) has been reported. Moreover, in our sample, 58% of all noted distal caries on second molars were associated with partially impacted third molars, 31% with fully erupted third molars, and only about 10% with fully impacted third molars. Clinically, it is evident that partially impacted third molars pose the greatest risk for second molar caries.^42^

For the maxillary third molars, our findings showed that distoangular impaction was most common, a pattern supported by the literature. In contrast, mesioangular impaction was by far the most prevalent angulation observed in the mandibular third molars.^23^^,^^32^^,^^43^^,^^44^ Sandhu and Kaur, along with Quek et al, similarly emphasised that mesioangular impaction was most frequently observed in the mandible, which could increase surgical difficulty. Some studies have found a predominance of vertical position in the maxilla.^24^^,^^45^ In addition, the predominance of vertical or distoangular positions in maxillary third molars may be related to the anatomical structures in the maxilla, such as the sinus cavity and the height of the jawbone.

Our study further demonstrated that the angulation is strongly tied to eruption status. Partially erupted teeth were mostly mesioangular, which makes sense because a tooth tilting forward will often partly emerge. Completely impacted teeth in the maxilla were mostly distoangular, which might be teeth that never had a chance to erupt downward and forward. This again is consistent with how one might clinically judge whether an impacted tooth has the potential to erupt or not: a mesioangular lower third molar with some crown exposed is likely in a holding pattern, whereas a distoangular upper third molar might be dormant but also unlikely to ever come into occlusion properly.

Our root morphology analyses have revealed that straight root form is the most common, followed by curved roots, with dilacerated roots occurring less frequently. Similar patterns were observed in Al-Madani et al’s study,^33^ classified as straight (53.9%), curved (28.7%) and dilacerated (5.7%). In Africa, Bokindo's study categorised root morphology as straight and dilacerated, finding curved and straight roots in similar proportions among impacted and erupted teeth.^46^ Our study shows a lower overall dilaceration rate of 9.43% (encompassing all jaw and tooth positions). This difference may be attributed to varying root development dynamics between jaws (maxilla/mandibula), sample size differences and discrepancies in the ‘root morphology evaluation criteria’ between the relevant groups. Indeed, Bokindo et al's exclusive focus on the mandible and acceptance of wider-angle ranges in dilaceration classification may be factors that increase their reported rate. Our study, by examining third molars in both jaws collectively, may show a lower average due to the relatively less frequent occurrence of dilaceration or ‘root curvature’ cases in the maxilla.

In our study, darkening of the root was the most prevalent sign. This finding is consistent with its recognised importance as a primary indicator of potential nerve involvement, as noted across numerous studies. However, reported frequencies for this sign vary widely, ranging, for example, from 6.05% in Rood and Shehab's prospective work^19^ to over 30% in other populations.^47^, ^48^, ^49^, ^50^ Notably, narrowing of the mandibular canal itself was the second most common sign in our sample, occurring in 24.27% of teeth. This prevalence is considerably higher than reported in several other studies,^19^^,^^48^^,^^49^ suggesting this could be a particularly relevant clinical indicator within our specific patient cohort. Other critical signs, such as interruption of the canal's white line and diversion of the canal, also exhibited varied prevalence compared to international findings. These discrepancies highlight population-specific anatomical variations and emphasise the clinical necessity of diligently identifying all such radiographic markers for thorough preoperative risk assessment and meticulous surgical planning to minimise potential nerve injury.

The findings of this study have noteworthy potential to inform clinical decision-making regarding the management of third molar teeth, particularly within the Kazakh population. The high prevalences of impaction and agenesis observed here highlight the importance of early and routine radiographic screening, especially for individuals aged 15 to 20 years, to detect third molar anomalies at an optimal stage. Such proactive screening could facilitate both the prediction of potential complications and the development of preventive strategies. By elucidating the prevalence of specific radiographic markers – including third molar angulation, eruption status and associated pathologies – and their relationship to the IAC, our results can help clinicians refine risk-assessment models and contribute to more personalised treatment planning.

This study has some limitations to acknowledge. First, it is cross-sectional and retrospective, relying on radiographic records. We did not have longitudinal follow-up to see which partially impacted teeth eventually erupted or were extracted, and what the long-term outcomes for adjacent teeth were. Second, although the sample size was substantial, participants were those who underwent panoramic radiography, for orthodontic assessment, wisdom-tooth evaluation, or routine check-ups, which may introduce selection bias towards individuals with third molar concerns. Nevertheless, because we enrolled all eligible patients across a broad clinical spectrum, we consider the cohort a reasonable proxy for young adults seeking dental care in our region. Lastly, our findings apply only to individuals aged 15 to 30 years; older adults, who may exhibit different patterns following tooth loss or extraction, were not represented. Despite these constraints, the study offers valuable baseline data on third molar prevalence and patterns within the Kazakh population.

## Conclusion

This study provides the first comprehensive analysis of third molar development in the Kazakh population, revealing a complex pattern of impaction and agenesis. With a high prevalence of third molar impaction (68.0%) and agenesis (37.28%), the research highlights significant variations influenced by gender, age and anatomical location.

The high prevalence of third molar impaction and agenesis in the Kazakh population underscores the need for region-specific clinical guidelines and management protocols. The findings highlight significant gender, age and anatomical location variations in the impaction patterns and associated pathologies. Early radiographic screening and diagnosis, especially in adolescents and young adults, can significantly influence clinical outcomes by preventing future complications associated with impacted third molars. While providing crucial baseline data, the study also calls for future longitudinal research to further understand the genetic and environmental factors influencing third molar development in this population.

## Conflict of interest

The authors declare that they have no known competing financial interests or personal relationships that could have appeared to influence the work reported in this paper.
